# Prevalence and Clinical Significance of Incidental Findings in the Maxillofacial Complex of Adolescent Orthodontic Patients: A Retrospective Cone Beam Computed Tomography Analysis

**DOI:** 10.7759/cureus.47480

**Published:** 2023-10-22

**Authors:** Lily Etemad, Shivam Mehta, Alan G Lurie, Aditya Tadinada

**Affiliations:** 1 Orthodontics, Siddiqui Orthodontics, Ann Arbor, USA; 2 Orthodontics, Marquette University School of Dentistry, Milwaukee, USA; 3 Oral and Maxillofacial Radiology, University of Connecticut School of Dental Medicine, Farmington, USA

**Keywords:** adolescent orthodontics, cone-beam computed tomography (cbct), 3d imaging, orthodontics, incidental findings

## Abstract

Objectives: The aim of this study was to determine the prevalence and severity of incidental findings in the maxillofacial complex of orthodontic patients imaged with cone beam computed tomography (CBCT) and assign those findings an appropriate clinical significance.

Methodology: Incidental findings (IF) were identified in 250 CBCT scans of adolescent orthodontic patients (aged 13-18 years) with a large field-of-view and categorized based on their anatomic location and placed into one of six subgroups based on anatomic region: i) sino-nasal, ii) dentoalveolar, iii) nasooropharyngeal airway, iv) temporomandibular joint, v) neck, vi) calcifications, and vi) miscellaneous findings. Additionally, findings were assigned a clinical significance score based on severity on a scale of mild, moderate and severe. Mild IF was defined as an IF that does not require any further investigation or referral. Moderate IF was defined as an IF that has the tendency to become clinically significant and should be observed periodically. IFs that warrant further investigation and/or intervention were designated as severe.

Results: The percentage of IFs in sino-nasal and dento-alveolar regions were 44.7% and 19.1% respectively. The percentage of IFs with mild, moderate, and severe clinical significance were 27%, 72%, and 1%, respectively. Out of the IFs involving calcifications, 80.8% were stylohyoid calcifications and <1% were cranial cavity IFs such as petroclinoid calcifications and falx cerebri calcifications. Among the sino-nasal findings, 1.2% were identified as severe.

Conclusion: The sino-nasal region had the highest frequency of IFs. Understanding the prevalence of incidental findings and its clinical relevance is important for clinicians to allow for appropriate monitoring and timely treatment of patients.

## Introduction

Cone beam computed tomography (CBCT) provides a three-dimensional (3D) assessment of the maxillofacial structures and is being increasing used in the field of orthodontics for diagnosis and treatment planning. The major applications of CBCT in orthodontics include but are not limited to diagnosis of skeletal and dental malocclusions, visualization of impacted teeth, airway analysis, treatment planning, and evaluation of the temporomandibular joint (TMJ) [1,2]. 

Incidental findings (IFs) are defined as any and all findings detected by an imaging modality that are unrelated to the clinical indication for which imaging is being performed [3]. IFs ranging from mild conditions to those with more severe ramifications have been reported with CBCTs [2,4]. Lateral cephalometric radiographs show a two-dimensional (2D) view of the 3D structures of the head and neck and thus, suffer from certain drawbacks such as superimposition of structures which limits the ability to clearly identify the IF from normal variation [5]. With CBCT, the ability to scroll through the different slices in a multiplanar mode enables a comprehensive evaluation of the anatomic structures in axial, coronal and sagittal view [5,6]. Thus, with the increasing usage of CBCT scans in orthodontics, more emphasis is being placed on investigating radiographic findings that were not so frequently encountered previously with 2D imaging modalities [7-15].

With a high number of investigations in head and neck imaging, more CBCTs are being recorded for orthodontic purposes [7,15]. Thus, it is important that orthodontists understand the incidence of IFs in CBCTs, and are knowledgeable in identifying IFs. Additionally, because CBCT spans the areas that may need an expert eye to identify the IF, collaborative work is highly recommended with maxillofacial radiologists for proper diagnosis of IFs and appropriate referrals for the management of IFs. Therefore, analysis of IFs in CBCT scans with a large field of view (FOV) recorded for orthodontic purposes is very important as it may show findings that may have a high clinical significance.

A few studies have shown that the incidence of clinically significant IFs with CBCT is not very high [15]. In contrast to such studies, certain other studies have shown that the incidence of IFs with CBCT to be in a very high range of 80-90% and have reported that more than 25% of such IFs either required treatment or referral [16]. Thus, there is an unsettled question in the current literature regarding the incidence of IFs in CBCT scans of orthodontic patients. Such conflicting findings warrant further investigation. One reason for such contrasting findings in the literature could be the difference in the FOV used for recording the CBCTs. 

We undertook this study with the aim to determine the prevalence of incidental findings in the maxillofacial complex in a sample of orthodontic patients imaged with a large FOV CBCT. Understanding the prevalence of incidental findings and its clinical relevance will allow the clinicians to identify IFs in their practice, appropriately monitor and referral of patients and also minimizing additional investigations for lesions not requiring any treatment.

## Materials and methods

This retrospective study evaluated a randomly selected sample of 250 CBCT scans of patients referred for orthodontic treatment in the Advanced Imaging facility at the University of Connecticut, School of Dental Medicine. Since this was a retrospective study that did not involve prospective enrollment of human subjects, an IRB waiver was approved for this study as a chart review by the University of Connecticut ethics committee. The inclusion criteria were scans of patients referred for orthodontic treatment, no significant metallic artifacts, no movement artifacts in the recorded CBCTs, no craniofacial syndromes, and scans with large FOV from bregma superiorly to menton inferiorly and from anterior nasal spine anteriorly to cervical spine posteriorly. All the scans were acquired with an Accuitomo CBCT machine (J. Morita Inc., Kyoto, Japan) using acquisition parameters of 90 kVp, 10 mA, 17.5 s; FOV 170 x 120 mm. The selected scans (age range 13 to 18 years) were de-identified by removing all HIPPA identifiers from the scans such as unique identification number, name, and date of birth. All the evaluators were blinded to any information regarding the CBCT scans including the medical or dental history.

The CBCT scans were evaluated using Invivo5 CBCT reconstruction software (Anatomage Inc., San Jose, CA, USA). The scans were evaluated in all three orthogonal planes and in volumetric rendering mode by a dental student (LE year 2) and two board-certified oral and maxillofacial radiologists (AT with 15 years of experience and AL with 40 years of experience). Extensive training was provided to the dental student for the identification of incidental findings. The raters were blinded to any clinical information other than it was for orthodontic evaluation as that was the inclusion criteria. Twenty-five CBCT scans were evaluated per session. Ten such sessions were undertaken for evaluation of a total of 250 CBCT scans. All the evaluators reviewed the CBCT scans on a split-screen dual-display monitor (HP Compaq LA2205wg) under standardized conditions of ambient light and sound. The evaluators had the complete control to analyze the CBCT scans in the three orthogonal planes as well as modify the contrast and histogram. The incidental findings were defined as findings discovered during the normal course of interpretation of a CBCT scan and unrelated to the stated diagnostic purpose of the acquired image volume. Findings were categorized based on their anatomic location and placed into one of six subgroups based on anatomic region: i) sino-nasal, ii) dentoalveolar, iii) naso-oropharyngeal airway, iv) temporomandibular joint, v) neck, vi) calcifications, and vii) miscellaneous findings. Additionally, findings were assigned a clinical significance score based on severity on a scale of mild, moderate and severe (Table 1).

**Table 1:** Frequency of incidental findings and clinical significance among designated anatomic regions (n=250) IFs: incidental findings, TMJ: temporomandibular joint

Incidental finding category	Percentage of IFs	Clinical significance
Sino-nasal	44.7	
Nasal septal deviation	54.0	moderate
Concha bullosa	17.6	moderate
Haller cell	4.4	moderate
Frontal sinus	39.2	moderate
Mastoid cells over TMJ	6.8	moderate
Mucous retention cysts	16.0	moderate
Mucosal thickening of maxillary sinus	47.6	moderate
Accessory ostium	7.6	mild
Pansinusitis	1.6	severe
Mucosal thickening of ethmoid & sphenoid sinus	2.0	moderate
Ostia blockage	1.2	moderate
Dentoalveolar	19.1	
Third molar	43.0	moderate
Supernumerary teeth	4.5	moderate
Missing teeth	11.6	moderate
Stafne's bone cavity	0.0	mild
Dilaceration	0.4	mild
Microdontia	0.8	mild
Root resorption	0.8	mild
Ectopic position	1.2	moderate
Torus mandibularis	0.4	mild
Odontogenic cyst	1.2	severe
Idiopathic osteosclerosis	0.8	moderate
Transposition	1.2	moderate
Pericoronitis	0.4	severe
Naso-oropharyngeal airway	20.0	
Enlarged adenoids	60.4	moderate
Enlarged tonsils	18.8	mild
Tonsil calcification	6.8	mild
Vascular calcification	0.8	mild
Temporomandibular joint	0.3	
Osteoarthritis	1.2	moderate
Neck	0.2	
Cervical osteoarthritis	0.8	moderate
Calcifications	14.5	
Stylohyoid ligament calcification	80.8	mild
Petroclinoid calcification	0.4	mild
Thyroid cartilage calcification	0.4	mild
Falx cerebri calcification	0.4	mild
Miscellaneous findings	1.2	
Segmental maxillary odontodysplasia	0.4	moderate
Odontoma	0.8	mild

Mild IFs were defined as IFs that do not require any further investigation or referral. Moderate IFs were defined as IFs that can have the tendency to become clinically significant and should be observed periodically. IFs that warrant further investigation and/or intervention were designated as severe IFs. The primary reasons for recording the CBCT for orthodontic purposes are listed in Table 2. The data were recorded in Microsoft Excel (Redmond, WA, USA) and statistical analysis was performed.

**Table 2:** Primary reason for recording CBCTs for orthodontic purpose CBCT: cone beam computed tomography

Primary reason	Number (n)	Percentage (%)
Impacted Canine	140	56
Other Impacted Teeth	23	9.2
Cleft	10	4
Third Molar	77	30.8
Total	250	100

## Results

The interrater and intrarater reliability was found to be high (>0.98). Of the 250 scans, 1084 incidental findings were identified among the six anatomic regions. The percentage observed findings located in the sino-nasal and dento-alveolar, representing 44.7% and 19.1% respectively. The number of findings in each anatomic region is presented in Table 1. 

**Figure 1:**
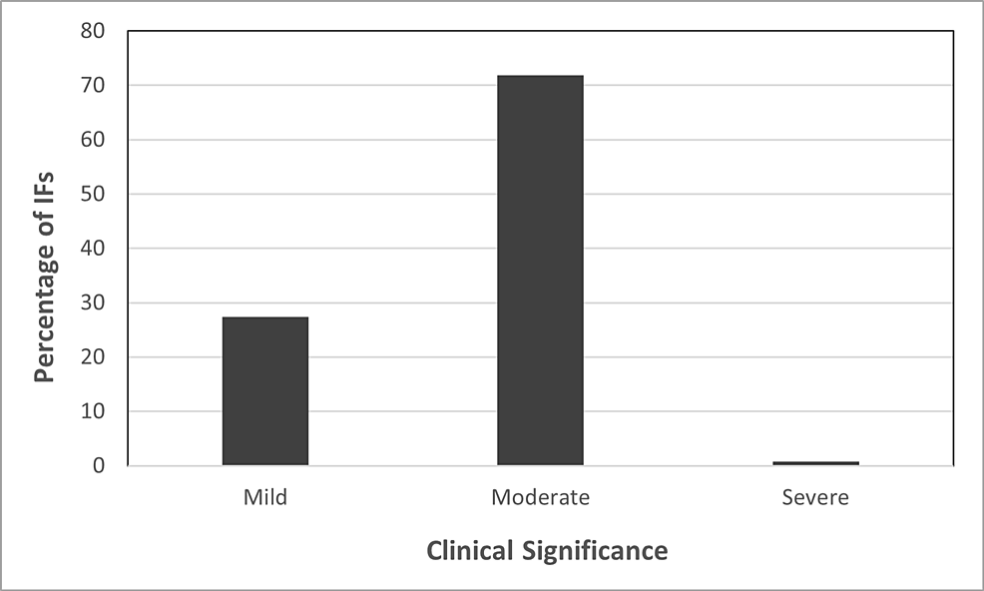
Frequency of incidental findings (IF) based on clinical significance of mild, moderate, and severe

Each incidental finding was given a clinical significance score of mild, moderate, and severe for staging of clinical significance. The percentage of incidental findings that were assigned a rating of mild was 27%, moderate was 72%, and severe was 1%, as presented in Figure 1.

Sino-nasal

**Figure 2:**
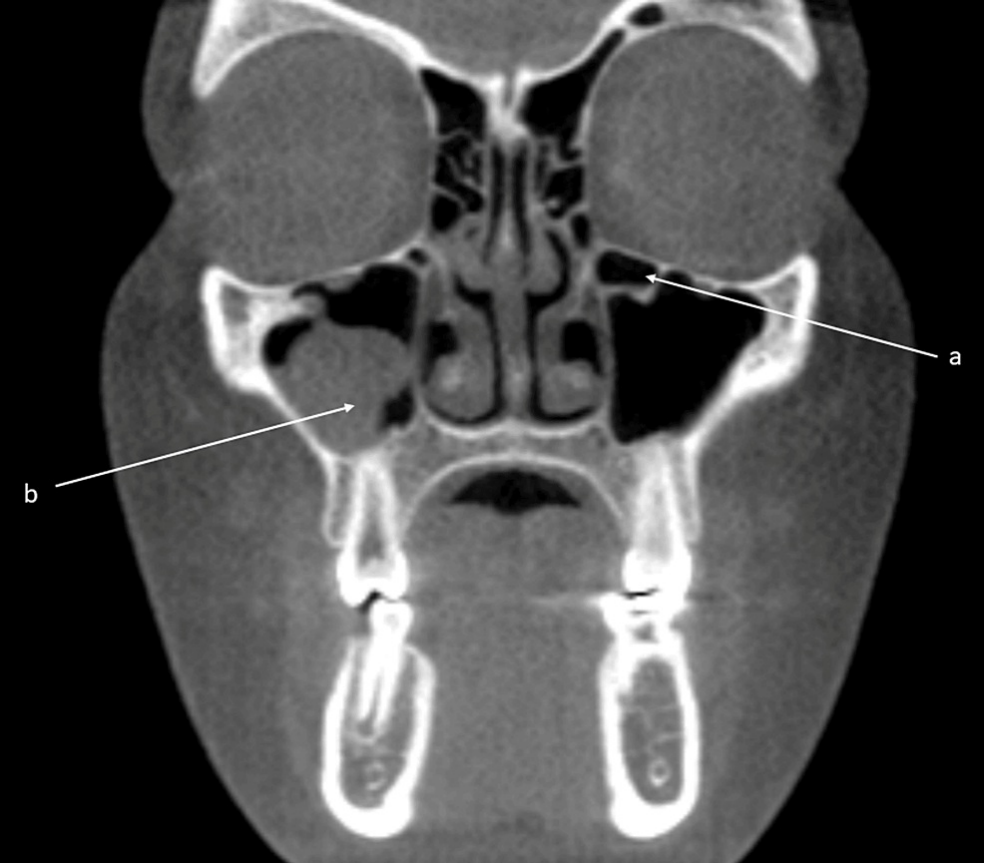
Coronal CBCT section reveals a Haller cell (a) along the left medial orbital floor. On the right side, moderate severity polypoidal mucoperiosteal swelling is present in the sinus floor (b). CBCT: cone beam computed tomography

**Figure 3:**
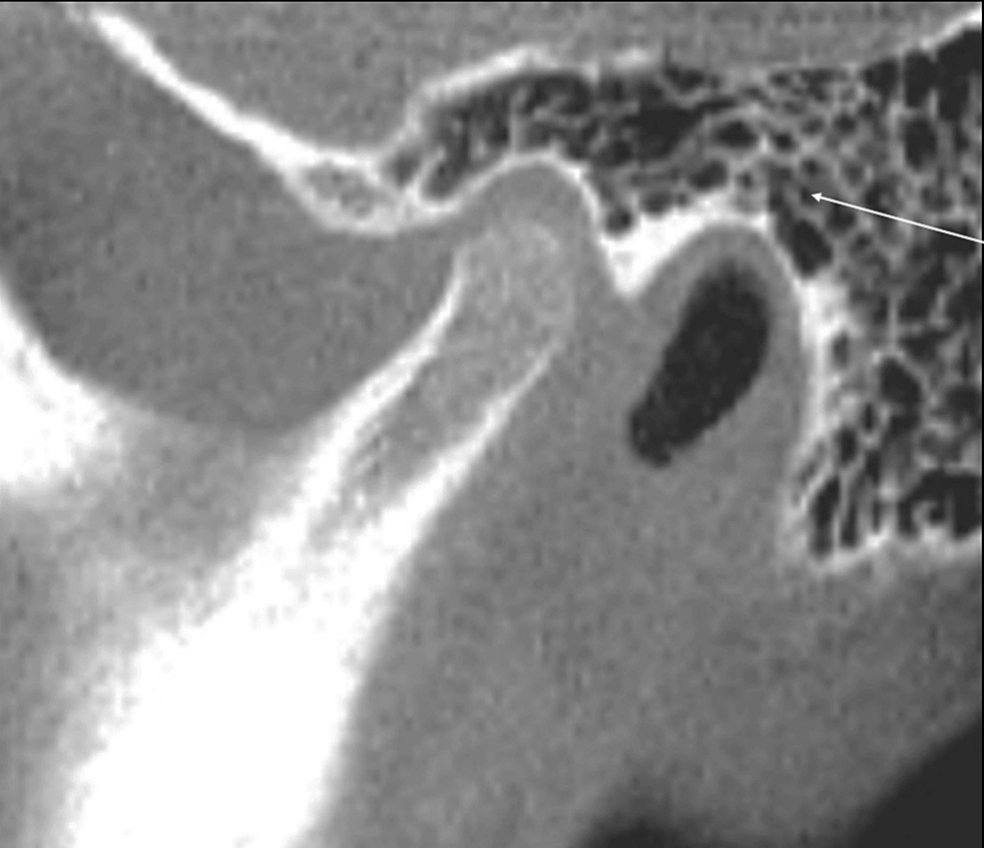
Sagittal CBCT section shows marked pneumatization of the temporal bone component of the TMJ by mastoid air cells CBCT: cone beam computed tomography, TMJ: temporomandibular joint

**Figure 4:**
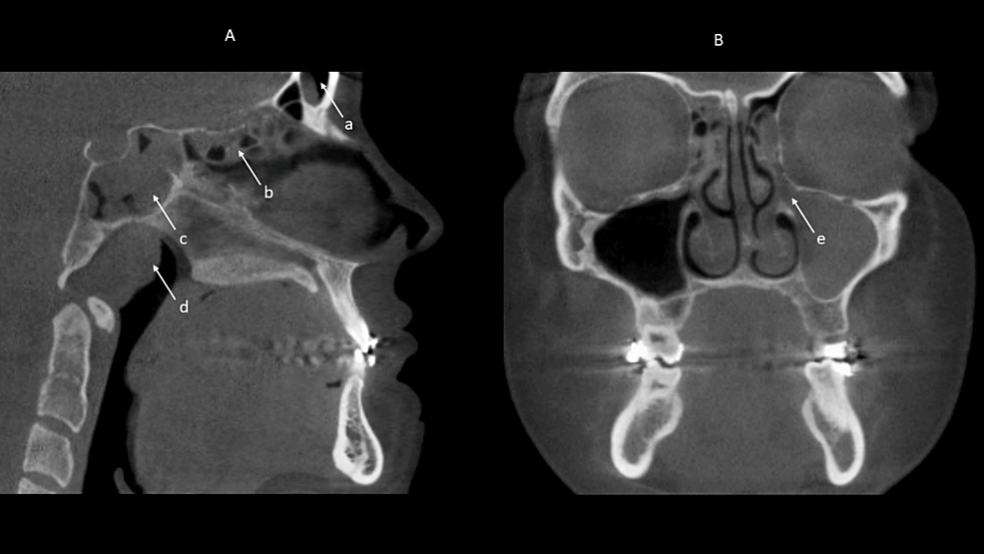
Various sinonasal abnormalities are shown in these CBCT sections from two different patients. A: Sagittal CBCT section shows enlarged mild to moderate mucosal thickening in frontal sinus (a), ethmoid air cells (b), and sphenoid sinuses (c). Enlarged adenoids (d) are also present. B: Coronal CBCT section shows a sino-nasal polypoid mass, a likely antrochoanal polyp (e), arising within left maxillary sinus passing through the ostium and into the left ethmoid air cells. There is associated marked enlargement of the ostium and slight elevation of the orbital floor. Mild to moderate mucosal swelling is also present in the right ethmoid air cells. CBCT: cone beam computed tomography

In the 250 scans, 44.7% of the IFs were identified in the sino-nasal anatomic region. Frequently identified findings were nasal septal deviation and mucosal thickening of the maxillary and frontal sinuses, representing 54.0%, 47.6%, and 39.2% of all findings respectively. The remainder of the findings within the sino-nasal region were concha bullosa (17.6%), mucous retention cyst (16.0%), accessory ostium (7.6%), mastoid cells in the temporal bone superior to the mandibular condyle or in the articular tubercle (6.8%), Haller cell (4.4%), mucosal thickening of other sinuses (2.0%), pansinusitis (1.6%), and ostium blockage (1.2%). Figures 2, 3, 4 depict common sino-nasal findings. Figure 4B depicts enlarged ostium and ethmoid sinusitis.

Dento-alveolar

The percentage of incidental findings observed in the dento-alveolar anatomic region was 19.1%. The most common findings were developing third molars (43%). Additional findings included missing teeth (11.6%), supernumerary teeth (4.5%), ectopic position of teeth (1.2%), transposition (1.2%), odontogenic cyst (1.2%), microdontia (0.8%), root resorption (0.8%), idiopathic osteosclerosis (0.8%), dilacerations (0.4%), and pericoronitis (0.4%).

Naso-oropharyngeal airway

Twenty percent of incidental findings were identified in the naso-oropharyngeal airway anatomic region. The most common finding was enlarged adenoids, representing 60.4% of all incidental findings. Enlarged tonsils (18.8%), tonsil calcification (6.8%), and vascular calcification (0.8%) were also identified. Figure 4A reveals enlarged adenoids and frontal, ethmoid, and sphenoid sinusitis.

Temporomandibular joint 

Osteoarthritis (1.2%) was observed within the TMJ anatomic region. 

Neck

Cervical osteoarthritis (0.8%) was observed within the cervical spine anatomic region.

Calcifications

**Figure 5:**
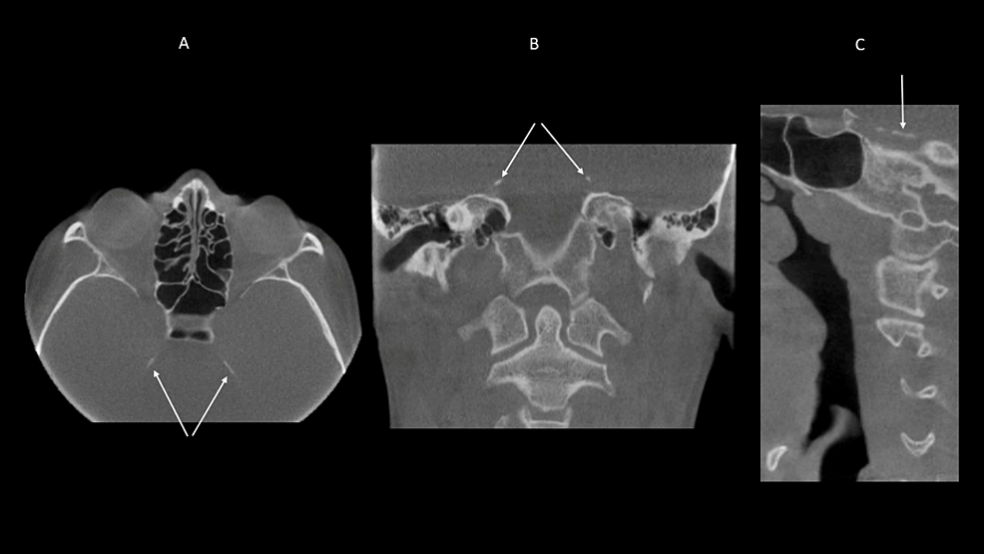
CBCT sections show bilateral, partial calcifications of the petroclinoid ligaments in axial (5A), coronal (5B) and sagittal (5C) planes CBCT: cone beam computed tomography

14.5% of incidental findings were identified as calcifications. A high occurrence of stylohyoid ligament calcification (80.8%) was present. The remainder of the findings were petroclinoid calcification (0.4%), thyroid ligament calcification (0.4%), and falx cerebri calcification (0.4%). Figures 5A, 5B, 5C reveal bilateral petroclinoid calcification.

Miscellaneous findings

Within the miscellaneous category, segmental maxillary odontodysplasia (0.4%) and odontoma (0.8%) were identified.

## Discussion

The primary reason for obtaining the CBCTs for orthodontic purposes has been described to be either impacted canines, orthodontic diagnostic records, or to examine bone pathologies [2,9,15,17]. Often, the purpose and the region of interest for recording the CBCT and the location of the incidental findings are widely different. Incidental findings in certain anatomical structures within the head and neck region such as the cranial cavity containing the brain, and the cervical areas which encompass important vascular structures are more critical as they can lead to serious clinical implications, if undiagnosed. The advantage of CBCTs with a large FOV, spanning the regions from cranium superiorly to the menton inferiorly and from anterior nasal spine anteriorly to cervical vertebrae posteriorly, is that the IFs in such critical areas can be identified. As compared to a small FOV CBCT used for certain cases such as impacted maxillary canines - which may show an IF of a mucous retention cyst within the maxillary sinus, a large volume FOV showing an IF of enlarged Sella may have more serious clinical implications.

Incidental findings within the cranial cavity are not very common [18]. Our study found that most IFs within the cranial cavity were uncommon with only one petroclinoid calcification and one falx cerebri calcification. Petroclinoid calcifications are age-associated calcifications in the petroclinoid ligaments. The low occurrence of petroclinioid calcifications in our study can be explained by the fact that in our study, the majority of the sample population consisted of young adolescents. Petroclinoid calcifications could either present in isolation or it could occur as a part of basal cell carcinoma syndrome and systemic fluorosis, and thus a detailed clinical history and examination would be useful in such cases [19,20]. The significance of calcifications within the cranial cavity is debatable. However, reports have indicated that such calcifications may be associated with conditions such as Alzheimer's disease, migraine, as well as stroke [21,22]. Orthodontists, dentists and maxillofacial radiologists can be the first ones to identify IFs in the CBCTs of orthodontic patients and thus, facilitate early diagnosis and management of such conditions. 

Mahdian et al. investigated the calcification of stylohyoid ligament in a patient population referred for dental implant treatment planning [23]. The authors reported that a significant number of patients (63%) show evidence of stylohyoid ligament calcification Our results in this study showed a higher occurrence of stylohyoid ligament calcification (80.8%) than that reported by Mahidan et al. [23]. This was an interesting finding as the population in our study was younger (adolescents) compared to the study by Mahdian et al. where the mean age of patients was 63.57 years. Thus, we can interpret that stylohyoid ligament calcification is not age-dependent as it is common even in adolescent patients undertaking orthodontic treatment. We further evaluated the data by assigning stylohyoid ligament calcification a clinical significance score of low, or no further investigation needed. Our study was based on radiographic interpretation only, without consideration of clinical information. If the patient however experienced cervical pain, sensation of a foreign body, dysphagia, or neurogenic symptoms such as tinnitus and visual disturbances due to pressure, then surgical intervention would be appropriate [23]. In such a case, with the presence of clinical symptoms the IF of stylohyoid ligament calcification would be given a higher clinical significance score.

The IFs in the sino-nasal area are significant to orthodontists and dentists because of the close proximity of the sino-nasal area to the dentoalveolar tissues. In our study, only four patients marked in the severe category for IFs were identified with pansinutis, which is lower than that reported by Edwards et al. [2]. Additionally, in our study, the prevalence of IFs for nasal septal deviation was found to be 58%. The nasal septum is an important structure in the nasal cavity as it helps in regulation of airflow through the nose and at the same time helps in providing shape and support to the dorsal and caudal aspects of the nose [24]. A deviation in nasal septum can lead to varying levels of nasal obstruction. Nasal septal deviation has been reported to be around 20% to 65%, which is similar to that found in our study [25]. Often patients with nasal septal deviation are asymptomatic and in such cases, monitoring the patients may be helpful [2,9,12]. However, in the presence of clinical symptoms and depending on the severity of such findings, preference may be given to managing the condition with a referral to the physician prior to conducting the orthodontic treatment.

The evaluation of the incidence of IFs provides important data regarding the utility of CBCT but it is only part of the total information. The clinically important question is how significant are the IFs in terms of their severity and clinical significance which might require further investigation or referral for treatment. In this study, we assigned a clinical significance score of mild, moderate, or severe to each incidental finding, based on the necessity for further investigation, intervention, and/or referral. The classification of IF based on clinical significance is beneficial to understand the prevalence of significant IFs that require early intervention and referral to specialists. For example, mucosal thickening of the maxillary sinus is an incidental finding observed in the sino-nasal anatomic region and its clinical significance is dependent upon severity of mucosal thickening. Mild mucosal thickening of the maxillary sinus is not clinically significant and does not warrant attention. Moderate mucosal thickening could progress to severe where it will be clinically significant, but only requires simple monitoring. Severe mucosal thickening may fill up the maxillary sinus and block the ostia, in which referral and further imaging of all sinuses are required [2].

CBCTs with large FOVs can lead to higher incidence of IF with significant clinical implications compared to CBCTs with small FOVs used for dental implant therapy. The inexperience of identifying the physiologic and pathologic processes in the CBCT at these locations can lead to undetected IF which may lead to inadvertent harm to the patient as well as clinical and legal implications. The evaluation of an orthodontic patient with CBCT utilizes a large FOV and consequently the scan may include several incidental findings. Clinicians should understand the potential liabilities and risks associated with the technology, specifically licensing and malpractice [26]. The clinician is responsible for the interpretation of the entire scanned set, which may be beyond the anatomic region for which the scan was taken [26-28]. For example, if a CBCT is recorded for orthodontic diagnosis and treatment planning, the clinician is responsible for not only the part of the image pertinent to an orthodontic analysis, but also for any findings that appear at other aspects on the image [28]. The American Academy of Oral and Maxillofacial Radiology (AAOMR) outlines that “if the interpreting clinician is not highly experienced in CBCT interpretation, appropriate referral is required to an oral and maxillofacial radiologist for review and that the entire volume must be interpreted regardless of the region of interest” [29]. 

A limitation of our study was that it was based on radiographic interpretation only, without clinical information. As a result, interpretation of clinical presentation and significance can be limited, specifically when related to sinus, airway, and TMJ findings, as symptoms play a big role in determining presence and severity of disease. However, the blinding of the clinical information provided an unbiased analysis of the CBCTs to identify the incidence and severity of IFs in the orthodontic patients from a radiographic perspective. Future studies should investigate the incidental findings with prior clinical information for greater accuracy in detecting and diagnosing disease. Another inadequacy is that the sample size was limited to a single location/center. Further studies are required on incidental findings in orthodontic patients from multiple centers and population groups to allow for a broader interpretation and understanding of the incidental findings.

## Conclusions

Several IFs are noted in CBCT scans of orthodontic patients; recognizing them and assigning them appropriate clinical significance is of value for intervention when necessary and triaging the patients. It is important to note that some incidental findings are anatomical variants and some are significant and require treatment. The knowledge regarding such incidental findings is important in recognizing when to intervene and when not to intervene. Therefore, it becomes important to note all the incidental findings in a CBCT scan. A complete evaluation of the entire CBCT scan is important to identify and classify the incidental findings according to their clinical significance to facilitate appropriate monitoring, referral and treatment of the patients in a timely manner.
